# Induction Strategies in Lung Transplantation: Alemtuzumab vs. Basiliximab a Single-Center Experience

**DOI:** 10.3389/fimmu.2022.864545

**Published:** 2022-06-01

**Authors:** Masashi Furukawa, Ernest G. Chan, John P. Ryan, Eric J. Hyzny, Lauren M. Sacha, Jenalee N. Coster, Joseph M. Pilewski, Elizabeth A. Lendermon, Silpa D. Kilaru, John F. McDyer, Pablo G. Sanchez

**Affiliations:** ^1^Division of Thoracic Surgery, Department of Cardiothoracic Surgery, University of Pittsburgh Medical Center, Pittsburgh, PA, United States; ^2^Department of Pharmacy, University of Pittsburgh Medical Center, Pittsburgh, PA, United States; ^3^Department of Pulmonology, University of Pittsburgh Medical Center, Pittsburgh, PA, United States

**Keywords:** lung transplant, induction immunosuppression therapy, basiliximab, alemtuzumab, acute cellular rejection

## Abstract

**Background:**

Induction therapy is used in about 80% of lung transplant centers and is increasing globally. Currently, there are no standards or guidelines for the use of induction therapy. At our institution, we have two induction strategies, basiliximab, and alemtuzumab. The goal of this manuscript is to share our experience and practice since this is an area of controversy.

**Methods:**

We retrospectively reviewed 807 lung transplants performed at our institution between 2011 and 2020. Indications for the use of the basiliximab protocol were as follows: patients over the age of 70 years, history of cancer, hepatitis C virus or human immunodeficiency virus infection history, and cytomegalovirus or Epstein-Barr virus (donor positive/ recipient negative). In the absence of these clinical factors, the alemtuzumab protocol was used.

**Results:**

453 patients underwent alemtuzumab induction and 354 patients underwent basiliximab. There were significant differences in delayed chest closure (24.7% alemtuzumab vs 31.4% basiliximab, p = 0.037), grade 3 primary graft dysfunction observed within 72 hours (19.9% alemtuzumab vs 29.9% basiliximab, p = 0.002), postoperative hepatic dysfunction (8.8% alemtuzumab vs 14.7% basiliximab, p = 0.009), acute cellular rejection in first year (39.1% alemtuzumab vs 53.4% basiliximab, p < 0.001). The overall survival rate of the patients with alemtuzumab induction was significantly higher than those of the patients with basiliximab induction (5 years survival rate: 64.1% alemtuzumab vs 52.3%, basiliximab, p < 0.001). Multivariate Cox regression analysis confirmed lower 5-year survival for basiliximab induction (HR = 1.41, p = 0.02), recipient cytomegalovirus positive (HR = 1.49, p = 0.01), postoperative hepatic dysfunction (HR = 2.20, p < 0.001), and acute kidney injury requiring renal replacement therapy (HR = 2.27, p < 0.001).

**Conclusions:**

In this single center retrospective review, there was a significant difference in survival rates between induction strategies. This outcome may be attributable to differences in recipient characteristics between the groups. However, the Alemtuzumab group experienced less episodes of acute cellular rejection within the first year.

## Introduction

The percentage of induction therapy for lung transplant recipients is increasing worldwide, with over 80% of patients receiving any form of induction therapy (1). The percentage of patients receiving an interleukin-2 receptor antagonist such as basiliximab has increased, and the percentage receiving anti-lymphocyte or anti-thymocyte globulin or alemtuzumab has decreased (1). Currently, there are no standards or guidelines for induction therapy. However, while still controversial, the use of induction therapy protocols can help reduce acute rejection and allow for minimization of maintenance immunosuppression in the perioperative period. Induction therapy mainly targets T cells, and T cells are considered the effector cells in cell-mediated rejection. Interleukin 2 receptor antagonists (basiliximab) are the most used induction therapies. Basiliximab is a monoclonal antibody targeting CD25, is generally well tolerated and has few side effects (1–3). Alemtuzumab is a humanized monoclonal antibody targeting CD52. CD52 antigen is found on T and B lymphocytes, natural killer cells, monocytes, and macrophages. Alemtuzumab induces cellular lysis and causes profound and prolonged suppression in T and B lymphocytes. Because of the body’s profound immunosuppressive response to alemtuzumab, patients may be given a reduced calcineurin inhibitor and antimetabolite exposure, and low dose steroid maintenance immunosuppression regimen in the immediate postoperative period (2–7). Our previous study showed that alemtuzumab induction had a survival rate comparable to that of thymoglobulin induction and may improve the prognosis of lung transplant, particularly graft survival, decreased frequency of acute cellular rejection and lymphocytic bronchitis, and reduced risk of chronic rejection compared to daclizumab induction or no induction (4).

The choice of induction protocol at our institution is based on several clinical factors, including donor and recipient cytomegalovirus (CMV) or Epstein-Barr virus (EBV) mismatch, cancer history, or older age (older than 70 years old). We are one of the few lung transplant centers that use both basiliximab and alemtuzumab induction and we would like to share our experience and practice since this continues to be an area of controversy.

## Patients and Methods

### Patients

**Figure 1:**
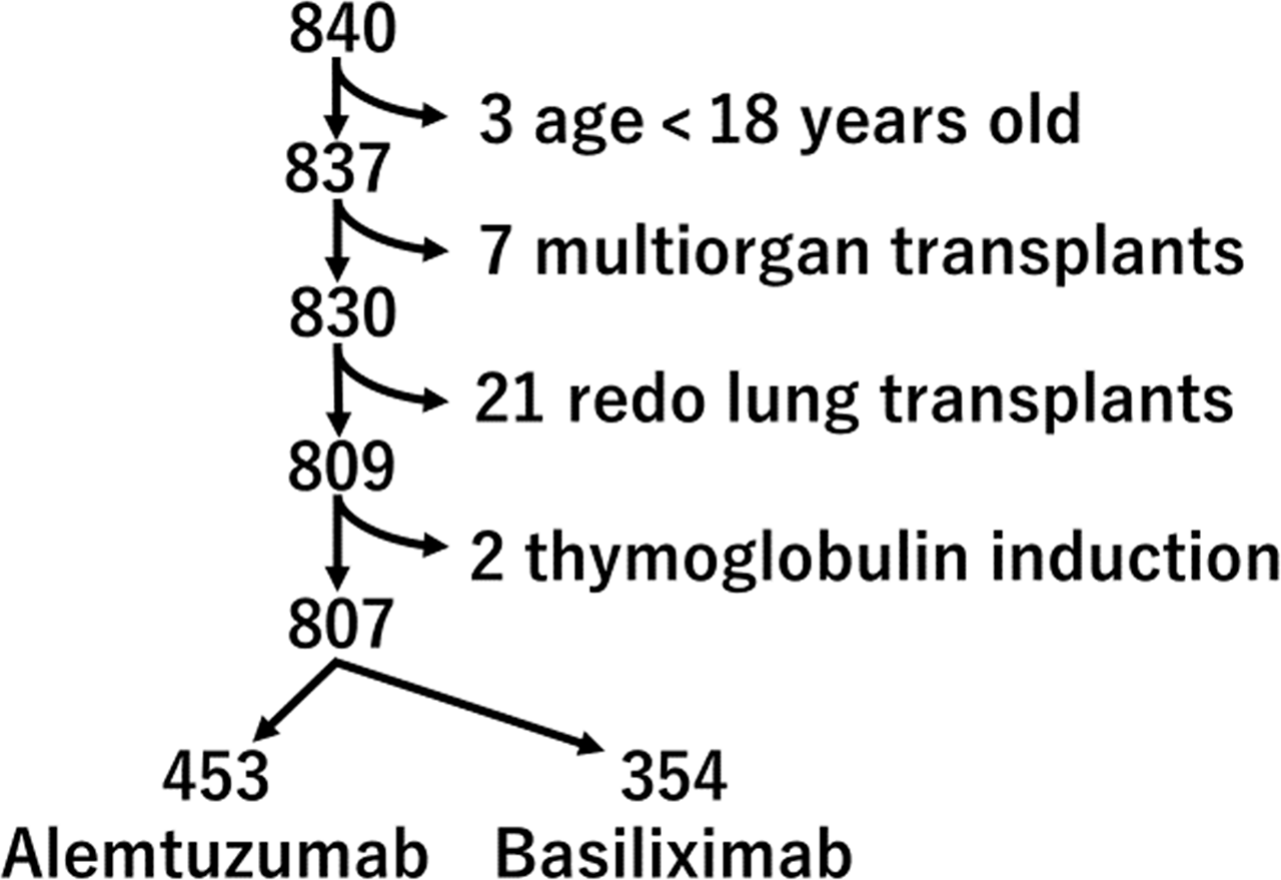
Algorithm of selection of study population.

We performed a retrospective analysis of all 840 lung transplant recipients at the University Pittsburgh Medical Center between 1/1/2011 and 12/31/2020. Exclusion criteria included age under 18 (n=3), multiorgan transplant [heart and lung transplants (n=6), lung and liver transplant (n=1)], re-do lung transplants (n=21), and thymoglobulin induction (n=2). The remaining 807 lung transplant recipients, which includes 453 alemtuzumab induction and 354 basiliximab induction, were part of this analysis **(** **Figure 1****)**. We assessed the preoperative and operative patient characteristics and postoperative course. The study protocol was approved by the institutional review board of the University Pittsburgh Medical Center.

### Induction Therapy Protocols

Choice of induction protocol was discussed in our multidisciplinary lung transplant meeting (pulmonology, thoracic surgery, critical care, transplant infectious disease and pharmacists, etc.) prior to listing. Indications for the use of the basiliximab protocol were as follows: patients over the age of 70 years, history of cancer, hepatitis C virus (HCV) or human immunodeficiency virus (HIV) infection history, and CMV or EBV mismatch (donor positive/ recipient negative). In the absence of these clinical factors, the alemtuzumab protocol was used. Currently, we are selecting induction according to this protocol, but in the early period of the observation period, it was determined by the discretion of the multidisciplinary committee and the primary physician, and a few cases deviate from the contemporary protocol. Our institutional protocol for immunosuppression is presented in **Table S1**.

### CMV Prophylaxis

Our institution employs a universal CMV prophylaxis strategy post-transplant. Recipients who are CMV high risk (donor seropositive and recipient seronegative) receive at least one year of oral valganciclovir 900 mg daily. In contrast, those who are moderate risk (donor seropositive or seronegative and recipient seropositive) receive at least six months of valganciclovir 450 mg daily. Patients are also routinely monitored for viremia by CMV DNA PCR approximately every 1-2 weeks for at least one year.

### CMV Treatment

Treatment for CMV infection is initiated when active viral replication is detected on DNA PCR with induction-dosed oral valganciclovir 900 mg twice daily or intravenous ganciclovir 5 mg/kg every 12 hours, at the discretion of the transplant pulmonologist and/or transplant infectious disease physician. Induction dosing of antiviral therapy is continued until clearance of viremia is documented by two consecutive negative DNA PCRs at least one week apart. Following induction dosed antiviral therapy, patients receive at least an additional three months of antiviral prophylaxis with oral valganciclovir or letermovir. CMV infection is also managed by the reduction in maintenance immunosuppression and/or incorporation of a mTOR inhibitor into the maintenance immunosuppression regimen, when appropriate.

### Biopsy and Rejection Treatment

Patients underwent transbronchial biopsy two weeks after lung transplant, either in the operating room or in our dedicated bronchoscopy suite, to determine the presence of acute cellular rejection. If grade 2 acute cellular rejection or higher was identified in the absence of any active infection, pulse dose i.v. (intravenous injection) methylprednisolone was given for three consecutive days.

### Statistical Analysis

Univariable analyses for continuous variables utilized Mann-Whitney tests, and categorical analyses were performed using Chi-squared test. Overall survival was analyzed with both the Kaplan-Meier method using a log-rank test and a multivariable-adjusted model using Cox regression. Univariable analyses were performed using SPSS (v. 27; Armonk, NY, USA). Survival and Cox regressions were performed using R (v. 4.0.2). Statistical significance was defined as p < 0.05.

## Results

There were some differences in preoperative characteristics among patients. We observed a significant difference in the distribution of recipient CMV positive (58.9% alemtuzumab vs 29.4% basiliximab, *p* < 0.001), CMV mismatch (9.5% alemtuzumab vs 60.2% basiliximab group, *p* < 0.001), and history of cancer (2.9% alemtuzumab vs 23.2% basiliximab, *p* < 0.001) (**Table 1**).

**Table 1:** Patient demographic and preoperative characteristics.

Variable	Alemtuzumab	Basiliximab	p value	Odds Ratio	95% CI
	n = 453	n = 354			
Age, Median (IQR)	60.23 (50.08 - 66.49)	60.16 (50.03 - 66.02)	0.58	-	-
Age 70 or older	39 (8.9)	37 (10.7)	0.42	1.22	0.76-1.95
Sex					
Female	201 (44.4)	144 (40.7)	0.29	1.16	0.88-1.54
Male	252 (55.6)	210 (59.3)			
Diagnosis					
COPD/Emphysema/BO	153 (33.8)	102 (28.8)	0.002	-	-
Pulmonary Fibrosis	162 (35.8)*	152 (42.9)*			
Suppurative	57 (12.6)	59 (16.7)			
Scleroderma	54 (11.9)*	19 (5.4)*			
Pulmonary Hypertension	9 (2.0)	10 (2.8)			
Occupational	15 (3.3)	6 (1.7)			
Other	3 (0.7)	6 (1.7)			
Lung allocation score, Median (IQR)	43.6 (34.6 - 66.6)	46.8 (36.1-67.9)	0.07	-	-
Body mass index, Median (IQR)	25.6 (21.4 - 29.3)	24.6 (20.9-28.7)	0.07	-	-
Waiting list time (days), Median (IQR)	50.0 (16.0 - 143.0)	46.5 (19.0-180.3)	0.29	-	-
Recipient CMV positive	267 (58.9)	104 (29.4)	< 0.001	0.29	0.22-0.39
Recipient EBV positive	436 (97.5)	334 (95.7)	0.15	0.56	0.26-1.24
HCV history	6 (1.3)	18 (5.1)	0.002	3.99	1.57-10.16
HIV history	0 (0.0)	6 (1.7)	0.005	0.43	0.40-0.47
CMV Mismatch	43 (9.5)	213 (60.2)	< 0.001	14.4	9.86-21.05
EBV Mismatch	10 (2.2)	14 (4.0)	0.15	1.82	0.80-4.16
Preoperative steroids	195 (43.0)	152 (42.9)	0.98	1	0.75-1.32
Preoperative hepatic disease	9 (2.0)	20 (5.6)	0.006	2.94	1.32-6.54
Oncology history	13 (2.9)	82 (23.2)	< 0.001	10.2	5.58-18.67
ECMO Bridge	36 (8.0)	34 (9.6)	0.41	1.23	0.75-2.01

^*^p < 0.05 post hoc. IQR, Interquartile range; COPD, Chronic obstructive pulmonary disease; BO, Bronchiolitis obliterans.

Intraoperative and postoperative variables also demonstrated significant differences in the need for delayed chest closure (24.7% alemtuzumab vs 31.4% basiliximab, *p* = 0.037), grade 3 primary graft dysfunction observed within 72 hours after lung transplant (19.9% alemtuzumab vs 29.9% basiliximab, *p* = 0.002), postoperative hepatic dysfunction (8.8% alemtuzumab vs 14.7% basiliximab, *p* = 0.009), acute cellular rejection in first year (39.1% alemtuzumab vs 53.4% basiliximab, *p* < 0.001) (**Table 2**).

**Table 2:** Operative and post-operative characteristics.

Variable	Alemtuzumab	Basiliximab	P value	Odds Ratio	95% CI
	n = 453	n = 354			
Lung transplant procedure					
Single	58 (12.8)	48 (13.6)	0.75	0.94	0.62-1.41
Bilateral	395 (87.2)	306 (86.4)			
Intraoperative support					
None	179 (39.5)	136 (38.4)	0.48	-	-
Cardiopulmonary bypass	140 (30.9)	123 (34.7)			
ECMO	134 (29.6)	95 (26.8)			
Total ischemic time, Median (IQR)	393.0 (335.5-466.5)	401.0 (342.0-458.0)	0.49	-	-
Operative time (hours:mins), Median (IQR)	8:02 (6:49-9:33)	8:10 (6:53-9:33)	0.54	-	-
Intraoperative blood transfusion, Median (IQR) [mL]	900.0 (300.0-1500.0)	900.0 (107.5-1800.0)	0.66	-	-
Delayed Chest Closure	112 (24.7)	111 (31.4)	0.037	1.39	1.02-1.90
Any grade 3 primary graft dysfunction Within 72 Hours	84 (19.9)	94 (29.9)	0.002	1.72	1.22-2.42
Postoperative ECMO	66 (14.6)	70 (19.8)	0.052	1.44	1.00-2.09
Acute kidney injury requiring renal replacement therapy	67 (14.8)	63 (17.8)	0.25	1.25	0.86-1.82
Stroke	16 (3.5)	10 (2.8)	0.57	0.79	0.36-1.77
Re-intubation	102 (22.5)	67 (18.9)	0.21	0.8	0.57-1.14
Bowel ischemia requiring bowel resection	17 (3.8)	10 (2.8)	0.47	0.75	0.34-1.65
Postop Hepatic Dysfunction	40 (8.8)	52 (14.7)	0.009	1.78	1.15-2.76
Hemothorax	54 (11.9)	42 (11.9)	0.98	1	0.65-1.53
Total intensive care unit stay (days), Median (IQR)	12.21 (4.0-19.0)	9.00 (4.0-18.0)	0.73	-	-
Total vent duration (days), Median (IQR)	3.88 (1.17-14.0)	5.00 (1.99-12.0)	0.38	-	-
Acute cellular rejection in First Year	177 (39.1)	189 (53.4)	< 0.001	1.79	1.35-2.37
Methylprednisolone (pulse dose)	176 (38.9)	149 (42.1)	0.35	1.14	0.86-1.52
Hydrocortisone (Stress dose)	133 (29.4)	90 (25.5)	0.22	0.82	0.60-1.13
Pneumonia	163 (36.0)	129 (36.4)	0.89	1.02	0.76-1.36
Wound Complication	85 (18.8)	72 (20.3)	0.58	1.11	0.78–1.57
Bronchiolitis obliterans syndrome	90 (19.9)	71 (23.5)	0.95	1.01	0.72–1.43

### Survival

**Figure 2:**
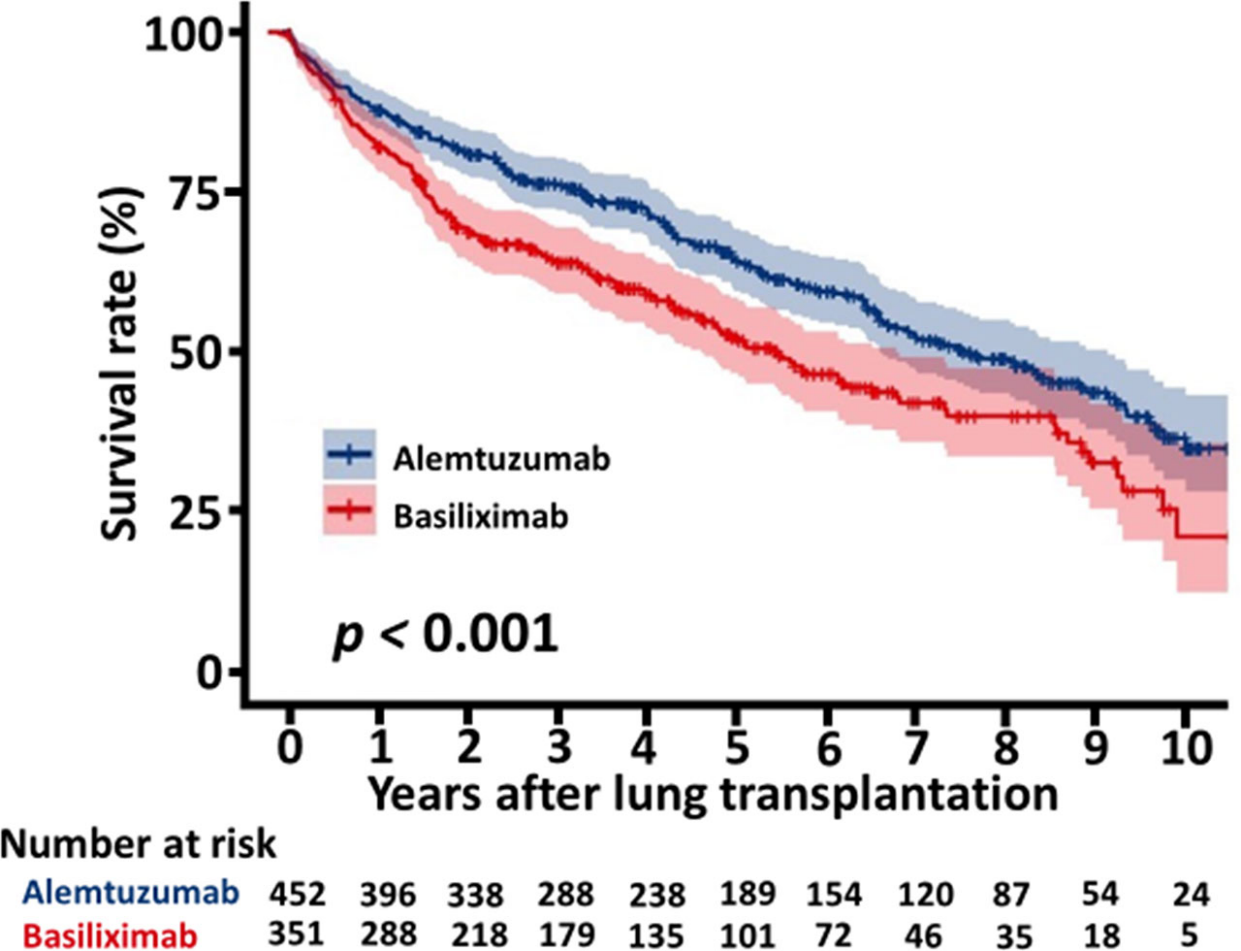
Kaplan-Meier curve for patients survival.

**Figure 3:**
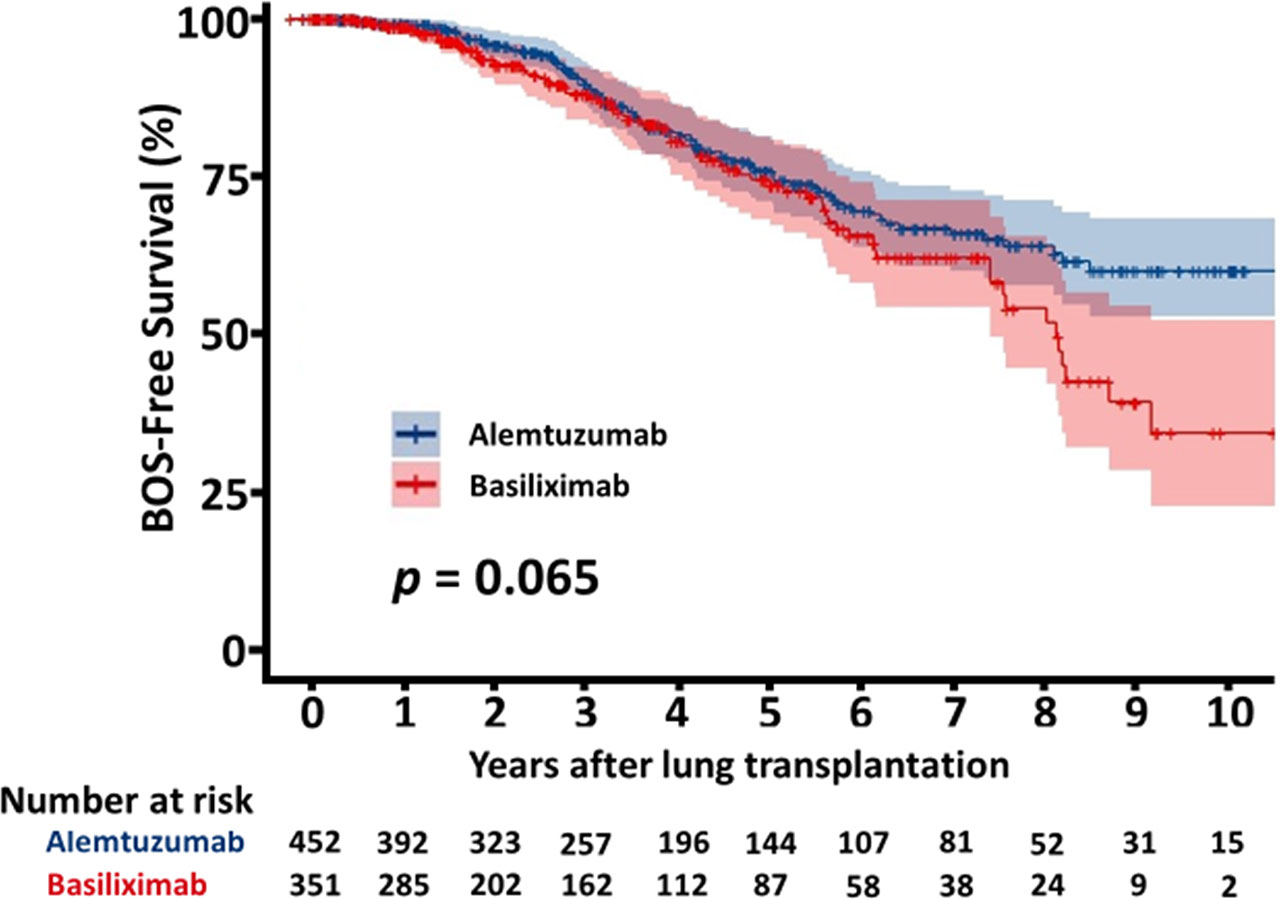
Kaplan-Meier curve for bronchiolitis obliterans syndrome (BOS) free survival.

The overall survival of the patients with alemtuzumab induction was significantly better than that of the patients with basiliximab induction (5 years survival rate: 64.1% alemtuzumab vs 52.3%, basiliximab, p < 0.001, **Figure 2**). **Figure 3** shows a comparison of bronchiolitis obliterans syndrome-free survival by Kaplan-Meier analysis. There was no significant difference between the two groups (*p* = 0.065). Cox regression analysis showed basiliximab induction (HR = 1.41, 95% confidence interval (CI) = 1.06-1.87, *p* = 0.02), recipient CMV positivity (HR = 1.49, 95% CI = 1.11-2.00, *p* = 0.01), postoperative hepatic dysfunction (HR = 2.20, 95% CI = 1.62-2.99, *p* < 0.001), and acute kidney injury requiring renal replacement therapy (HR = 2.27, 95% CI = 1.73-2.98, *p* < 0.001) were independent risk factors for overall survival (**Table 3**). There was no significant difference in recipient cause of death (**Table 4**).

**Table 3:** Multivariable Cox regression predicting post-transplant survival.

	O.R.	95% C.I.	P value
Induction (Basiliximab)	1.41	1.06 - 1.87	0.02
Diagnosis-Obstructive	ref	ref	ref
Diagnosis-Pulmonary hypertension	0.75	0.42 - 1.35	0.34
Diagnosis-Suppurative	0.68	0.45 - 1.02	0.06
Diagnosis-Restrictive	1.02	0.78 - 1.34	0.89
Lung allocation score	1	0.99 - 1.00	0.24
Recipient CMV positive	1.49	1.11 - 2.00	0.01
CMV Mismatch	1.13	0.79 - 1.62	0.51
Oncology history	1.2	0.85 - 1.68	0.3
Any grade 3 primary graft dysfunction Within 72 Hours	1.21	0.93 - 1.58	0.16
Treatment acute cellular rejection in First Year	0.84	0.67 - 1.06	0.13
Postoperative hepatic dysfunction	2.2	1.62 - 2.99	< 0.001
Acute kidney injury requiring renal replacement therapy	2.27	1.73 - 2.98	< 0.001

Odds ratio for death are reported (OR>1 implies an increased risk of death).

**Table 4:** Cause of death.

Variable	Alemtuzumab	Basiliximab	P value
	n = 453	n = 354	
Cause of death, n (%)			0.48
Cardiovascular	18 (9.1)	16 (9.1)	
Infection	52 (26.4)	46 (26.1)	
Malignancy	28 (14.2)	14 (8.0)	
Primary graft failure	27 (13.7)	32 (18.2)	
PTLD	6 (3.0)	6 (3.4)	
Other	66 (33.5)	62 (35.2)	

## Discussion

There are few reports that directly compare alemtuzumab and basiliximab as induction therapy for lung transplant (2, 3, 8). In our study, we found that acute cellular rejection was significantly lower in the alemtuzumab group within the first year. There was a significant difference in survival between the two groups, with better survival in the alemtuzumab group compared to the basiliximab group. The rate of primary graft failure as a cause of death was not significantly different in the two groups. There was no significant difference in the incidence of bronchiolitis obliterans syndrome or bronchiolitis obliterans syndrome-free survival between the alemtuzumab group and the basiliximab group. We could not determine the cause of the difference in survival. This may primarily be due to intrinsic selection bias, since several recipient related factors conferred a higher risk profile to the patients in the basiliximab group: more patients with pulmonary fibrosis, HCV history, HIV history, and history of cancer. In the Cox regression model, survival was affected by basiliximab induction, recipient CMV positivity, postoperative hepatic dysfunction, and acute kidney injury requiring renal replacement therapy. Hepatic dysfunction and acute kidney injury reflect surgical complexity for these patients and a negative effect in their survival. In multivariate analysis, although recipient CMV positivity was a risk factor for death (OR 1.49, 95%-CI 1.11-2.0), CMV mismatch was not (OR 1.13, 95%-CI 0.79-1.62). This might be due to the differences in the baseline characteristics of the two groups, which were accounted for in the multivariate analysis. It is also possible that our universal treatment protocols for CMV may have contributed to CMV mismatch not being a risk factor for death. Basiliximab induction was a poor prognostic factor, but this is difficult to explain because of the several confounders in the baseline characteristics, such as diagnosis, history of cancer, and the number of patients over 70 years of age.

Whitson et al. used data from the United Network for Organ Sharing (UNOS), the United States of America organ procurement and transplantation network, and reported that basiliximab induction or alemtuzumab induction was associated with better survival than no induction, but they did not make a direct comparison between basiliximab and alemtuzumab (8). Furuya et al. also used UNOS data and reported that while basiliximab induction and alemtuzumab induction had better survival rates than those without induction, survival of the alemtuzumab induction group is similar to those of the basiliximab induction group (2). They also reported that alemtuzumab was associated with longer freedom from bronchiolitis obliterans syndrome compared with basiliximab or no induction. Whited et al. reported that in a direct comparison of alemtuzumab and basiliximab induction in a single-center, the alemtuzumab group had less acute cellular rejection at six months postoperatively and had similar infection and survival rates (3).

We have previously reported that the basiliximab induction was associated with higher bronchial dehiscence than the alemtuzumab induction (9). We believe that this may be largely due to the higher dose of methylprednisolone in the immediate postoperative period that our basiliximab protocol requires. We also previously reported that early post-transplant lymphoproliferative disease (PTLD) is associated with alemtuzumab induction, especially EBV-mismatched groups (10). Alemtuzumab is thought to cause stronger immunosuppression, and there was concern that it could increase malignancies and infections. In our study, there was no significant difference in infections such as pneumonia or wound infection. It may be important to note that although there were fewer patients with prior cancer history in the alemtuzumab group than in the basiliximab group, deaths from cancer were similar in both groups.

Several limitations of our study should be mentioned. First, this study was retrospective, and therefore the results are subjected to recall and reporter biases. Second, we report the results of a single-center analysis. Third, because the indications for the use of the two induction protocols are different, there may be unmeasured confounding variables associated with these indications that may have influenced the results. There are also numerous other confounding variables that may have significantly affected patient survival, including maintenance immunosuppression regimens, the incidence of acute cellular rejection beyond the first post-transplant year, the occurrence of antibody-mediated rejection, CMV infection, or disease, other infectious processes, and post-transplant malignancy.

However, all in all, our results suggest that alemtuzumab should still be considered as a viable and effective induction strategy with the potential to reduce acute cellular rejection.

## Data Availability Statement

The raw data supporting the conclusions of this article will be made available by the authors, without undue reservation.

## Ethics Statement

The studies involving human participants were reviewed and approved by University of Pittsburgh Institutional Review Board. The patients/participants provided their written informed consent to participate in this study.

## Author Contributions 

MF and PS contributed to the conception and design of the study. EC and EH organized the database. JR performed the statistical analysis. MF, PS, JC, JP, EL, SK, and JM participated and decided the choice of induction therapy. MF wrote the first draft of the manuscript. JR and LS wrote sections of the manuscript. All authors contributed to manuscript revision, read, and approved the submitted version.

## Conflict of Interest

The authors declare that the research was conducted in the absence of any commercial or financial relationships that could be construed as a potential conflict of interest.

## Publisher’s Note

All claims expressed in this article are solely those of the authors and do not necessarily represent those of their affiliated organizations, or those of the publisher, the editors and the reviewers. Any product that may be evaluated in this article, or claim that may be made by its manufacturer, is not guaranteed or endorsed by the publisher.

## Supplementary Material

Supplementary Table 1 Immunosuppression regimens.

## Abbreviations

CI, confidence interval; CMV, Cytomegalovirus; EBV, Epstein-Barr virus; ECMO, extracorporeal membrane oxygenation; HCV, hepatitis C virus; HIV, human immunodeficiency virus; i.v., Intravenous injection; PTLD, post-transplant lymphoproliferative disease; UNOS, United Network for Organ Sharing.
